# Conservation Priorities for *Prunus africana* Defined with the Aid of Spatial Analysis of Genetic Data and Climatic Variables

**DOI:** 10.1371/journal.pone.0059987

**Published:** 2013-03-27

**Authors:** Barbara Vinceti, Judy Loo, Hannes Gaisberger, Maarten J. van Zonneveld, Silvio Schueler, Heino Konrad, Caroline A. C. Kadu, Thomas Geburek

**Affiliations:** 1 Headquarters, Bioversity International, Rome, Italy; 2 Regional Office for the Americas, Bioversity International, Cali, Colombia; 3 Department of Genetics, Federal Research and Training Centre for Forests, Natural Hazards and Landscape (BFW), Vienna, Austria; 4 Department of Biochemistry and Biotechnology, School of Pure and Applied Sciences, Kenyatta University, Nairobi, Kenya; 5 Faculty of Bioscience Engineering, Ghent University, Gent, Belgium; CNR, Italy

## Abstract

Conservation priorities for *Prunus africana*, a tree species found across Afromontane regions, which is of great commercial interest internationally and of local value for rural communities, were defined with the aid of spatial analyses applied to a set of georeferenced molecular marker data (chloroplast and nuclear microsatellites) from 32 populations in 9 African countries. Two approaches for the selection of priority populations for conservation were used, differing in the way they optimize representation of intra-specific diversity of *P. africana* across a minimum number of populations. The first method (S1) was aimed at maximizing genetic diversity of the conservation units and their distinctiveness with regard to climatic conditions, the second method (S2) at optimizing representativeness of the genetic diversity found throughout the species’ range. Populations in East African countries (especially Kenya and Tanzania) were found to be of great conservation value, as suggested by previous findings. These populations are complemented by those in Madagascar and Cameroon. The combination of the two methods for prioritization led to the identification of a set of 6 priority populations. The potential distribution of *P. africana* was then modeled based on a dataset of 1,500 georeferenced observations. This enabled an assessment of whether the priority populations identified are exposed to threats from agricultural expansion and climate change, and whether they are located within the boundaries of protected areas. The range of the species has been affected by past climate change and the modeled distribution of *P. africana* indicates that the species is likely to be negatively affected in future, with an expected decrease in distribution by 2050. Based on these insights, further research at the regional and national scale is recommended, in order to strengthen *P. africana* conservation efforts.

## Introduction

The identification of priority sites for conservation action remains a central issue in the implementation of conservation interventions, due to the fact that resources are usually limited and competition for land is high. Different approaches for making conservation choices when resources are scarce have been described previously [1]–[4]. Those currently proposed in the literature are based on a combination of different criteria, including measures of diversity, assessments of risk status and conservation costs [5], applicable at the level of vegetation type, species, or molecular diversity [6]–[10].

Despite the recognized importance of evolutionary processes, they have often been excluded in conservation assessments and planning, which are more frequently based on species richness, rather than intra-specific diversity indicators [8], [11]; rare are the cases in which morphological and demographic variables have been integrated with genetic parameters to define the appropriate location of conservation units of threatened species [12]. In the case of multi-taxon approaches, evidence shows that those based on species richness fail to represent rare, threatened, or genetically distinct species [13]. The use of range-weighted matrices is an example of an approach that accounts for range sizes and the higher probability of extinction for species that are geographically restricted, compared to congeners with wide distribution [14].

Knowledge of the distribution of genetic diversity adds valuable information to support conservation efforts because the capacity of a species to adapt to changing environmental conditions depends on its heritable variation, which allows evolutionary processes to take place [15]. In the absence of data on the distribution of a species’ genetic variation, sites for conservation could be selected more or less uniformly throughout the species’ natural range [16], if the environmental conditions are relatively uniform or if they follow a continuous gradient. Genecological approaches have been used [17], [18], based on the assumption that tree genetic variation follows some of the patterns of ecological variation [19]. However, patterns are species-specific; species react differently to environmental gradients. The correlation between genetic diversity and species richness is controversial [20], and species diversity and phylogenetic diversity have different patterns of spatial distribution [13]. Thus, when genetic data are available they provide more precise information for decision-making [21]. They can also support assessments of extinction risks [22].

Some key questions continue to pose challenges. Determining the number of populations, and the number of individuals within a population, that are needed to capture useful genetic variation in a species, or within part of a species’ range, is not simple and there are many estimates in the published literature [23], [24], which, in some cases, are species-specific [25]. Another question relates to what criteria should be used, based on genetic parameters, to define priorities (mean number of alleles per locus, percentage of polymorphic loci, etc). In addition, the question about whether conservation emphasis should be higher in peripheral or central populations, is not resolved and species-specific [26]–[31]. Generally, diversity is higher in populations located in central parts of the distribution range. However, peripheral populations often have valuable adaptive traits that are specific to marginal environments [32], and species that have highly dispersed, isolated populations may not have a recognisable centralised distribution. Furthermore, issues related to population size matter. Small populations that are geographically and genetically isolated from each other have been shown to lose diversity more rapidly than larger populations or small populations that are linked by gene flow [33].

The concept of the evolutionarily significant unit, defined as “a population that merits separate management and has a high priority for conservation” [34] has been proposed [35]. However, its identification is based on several parameters (e.g., morphological and phenological traits, biochemical and molecular markers), making the approach difficult to adopt in practice, especially at large spatial scales. It is known that a research–implementation gap exists in conservation planning, for reasons that include the lack of dialogue between scientists and managers of natural resources [36]; the gap can be widened if conservation methods are perceived as too costly and sophisticated.

Finally, conservation priorities have usually been defined based on a static snapshot of the current situation, but it is increasingly recognized that estimates of species vulnerability to global environmental changes should be incorporated in conservation planning decisions. This can be assessed in terms of predicted loss of climatically suitable areas for a particular species [37], and more recently looking separately at sensitivity, and adaptive capacity [38].

Among the African indigenous tree species, *Prunus africana* (also known by its previous name, *Pygeum africanum,* Hook f.), with a broad but disjunct distribution across Afromontane regions, has been studied due to its economic importance: occurrence data from herbaria offer wide coverage and recently genetic data derived from a continent-wide collection have been made available. Over the past several decades, products from *P. africana’*s bark extracts have been the most widely exported of any African tree species for medicinal purposes, contributing to its overexploitation. Due to the threats to the species, mainly posed by overexploitation, but also by agricultural expansion and expected environmental changes, the need for a conservation strategy has been highlighted [39]; the knowledge that has become available, such as occurrence data and genetic information, is an ideal base to develop a continent-wide conservation strategy, starting with the identification of key conservation sites.

The current study builds on molecular marker data for *P. africana* generated as part of two studies by Kadu et al. [40], [41], which showed a clear phylogeographic pattern for the species, suggesting an early split of ‘east’ and ‘west’ types during southward migration, followed by more recent splitting events among eastern populations. These findings confirm the results from earlier pilot studies based on molecular markers showing the strong partitioning of genetic variation of *P. africana* across geographical distance, due to its wide but disjunct distribution in Afromontane forest islands [42]. All these results suggest that a considerable number of sites may be required for effective conservation of the genetic variation within the species.

We address a series of issues related to the definition of a conservation strategy for *P. africana,* focusing primarily on the identification of priority populations for conservation. The formulation of a conservation strategy requires understanding a species’ spatial distribution. We generated a distribution map of *P. africana* based on predictions of the potential species’ distribution from records and environmental data from the same sites, using GIS (Geographic Information System) [43]–[46]. The resulting map indicates areas of high and low probability of occurrence based on the species’ current ecological niche (a review of spatially explicit methods used by biogeographers can be found in [46]).

We adopted the number of alleles (i.e., allelic richness) as a key measurement to determine priorities in the conservation of the genetic diversity in *P. africana*. This is a very informative parameter, as the number of alleles per locus is dependent on effective population size, therefore a good indicator of past demographic changes that would have affected genes associated with adaptive traits as well as neutral markers [47]. This parameter is considered ideal especially for hypervariable markers such as nuclear microsatellites [48].

In order to define the location and number of populations needed to optimally conserve *P. africana*, we proposed an approach based on spatial analyses of genetic parameters and climatic variables. Geospatial methods can be applied to phylogeography [46] and can be used to carry out complex analyses that facilitate the selection of priority populations for conservation of genetic resources [49]–[51]. Accurate spatial information on the occurrence of a species combined with climatic variables has also proven useful for effective genebank management (e.g., definition of core collections, identification of collection gaps, etc.) [52]. Geospatial approaches have been adopted for the conservation *in situ* of species for which it is not possible to rely on *ex situ* conservation strategies. An example is that of important crop wild relatives, for which conservation can be achieved only by managing wild populations *in situ*, and which derive their properties from their adaptation to environmental conditions, favoured by evolutionary processes [53], [54].

More specifically, we proposed the selection of a core set of priority populations based on a combination of two methods: one aimed at maximizing genetic diversity and distinctiveness of the conservation unit with regard to climatic conditions (S1), the other at optimizing representativeness of the genetic diversity found throughout the species’ range (S2). Both approaches attempt to maximize the species’ evolutionary potential through the identification and conservation of populations with the highest possible levels of genetic variation. The assumption is that this variation will allow evolutionary processes to take place and foster adaptation (e.g., [55]–[57]). These results were obtained through user-friendly, GIS-based, free access software and constitute a first level in the decision making process to which, subsequently, economic and other considerations could be added [58].

Finally, we examined the conservation status of the populations selected, based on the location of our proposed sites for conservation *vs.* existing protected areas, and the expected threats posed by changing environmental conditions, using spatial analyses to build projected impacts of climate change on the distribution of the species [59].

### *Prunus africana*

*P. africana* is of great commercial interest due to the preparation of medicinal products from its bark, used to treat benign prostatic hyperplasia. The species also plays an important ecological role, providing food and home for pollinators and rare fauna, and supporting vascular and non-vascular canopy epiphytes [60]. In addition to local use and trade, the collection and processing of the bark has created economic opportunities for rural communities. A vast literature describes the various uses and its importance in the preparation of medicinal products from its bark, marketed internationally [61]–[66].

The species has a broad but highly fragmented distribution, spanning the African continent from South Africa to Ethiopia and west to Cameroon, but is limited to montane regions where it can be locally common [67]. Genetic considerations are particularly relevant for the management and conservation of *P. africana*, due to its close association with montane regions and low colonization potential [68]. The species has hermaphrodite flowers pollinated by insects. Although self-fertile, it is usually outcrossing; fruits are dispersed by birds and monkeys [69]. Unsustainable debarking of *P. africana*, disproportionately affecting and ultimately causing the death of large, reproductively mature individuals [70] is likely to cause reduced seed dispersal and gene flow, increasing isolation and reducing viability of existing populations. *P. africana* has been reported as a pioneer [71] or early successional species, associated with forest edges and disturbance [72].

Typically, the species is found where the annual temperature range is 18–26°C, mean annual rainfall ranges from 890 to 2,600 mm, and at an elevation between 900 and 3,400 m, with increasing elevation range towards lower latitudes. Its distribution range is limited by high temperatures and by insufficient precipitation during the warmest months [39]. Moist conditions could trigger infestation of powdery mildew and occurrence of a stem borer, whose presence is indicated by resin exuded through small bore holes [67]. Stem borers seem to be a serious problem when the species is planted in lowland areas, as observed in Cameroon [66].

Over the past 40 years, *P. africana* bark harvest has shifted from subsistence and local use to large-scale commercial use for international trade. Studies on the impacts of wild harvest on *P. africana* populations have shown that the practices adopted and the quantities extracted are not sustainable [62], [70]. Because of concerns for the sustainability of the trade, the species has been assigned a vulnerable conservation status on the IUCN Red List, and was proposed by Kenya for CITES in 1994 [73], then listed in 1995 in CITES Appendix II. Import of the bark from Cameroon into the European Union was banned from November 2007 to December 2010, when CITES lifted the ban subject to a reduced quota of 150,000 kg for 2010 and 2011 compared to two million kg of bark in 2005, reduced to one million kg in 2008 [74]. In various African countries, policies have been established aiming to ensure the sustainable management of forests that contain *P. africana* stands. However, enforcement issues and control problems persist, and there is considerable urgency to identify and implement sustainable management options, including conservation and appropriate domestication measures.

## Materials and Methods

### Population Sampling and Data Source

Two main datasets on *P. africana* were used in this study: a) a set of genetic data from 32 populations collected in the near range-wide study by Kadu et al. [40], [41], and b) a set of 1,500 georeferenced observations obtained from various sources (of which the 32 populations above are a subset).

The *P. africana* georeferenced chloroplast (cpSSRs) and nuclear microsatellites (nSSRs) consisted of: 7 chloroplast DNA loci from 582 individual trees; 6 nuclear loci from 484 individual trees. The DNA was isolated from leaf samples that were collected during 2007 and 2008 in natural stands (ie, not planted) in 9 African countries. These are from 32 accurately georeferenced populations (Table 1), spatially distributed in order to cover as extensively as possible the species range, across Afromontane forests (it was not possible to gain access for sampling purposes to Ethiopia and Angola). The sites were chosen based on a) their degree of isolation (selecting sites on well-separated mountain chains across the African continent), b) different ecological conditions (including geological substrate), c) availability of logistical support for sampling, d) expected size of the populations (i.e., populations expected to be too small were avoided *a priori*). Samples were collected by research partners from local institutions in different countries as indicated in Kadu et al. [40], [41].

**Table 1:** *Prunus africana* populations of conservation priority based on the first selection method proposed (S1).

			cpSSR				nSSR				Bioclim 2.5
N	Name of population	Country	n	Genetic distance Cluster	Haplotype richness (ranking)	LCA (ranking)	n	Genetic distance Cluster	Allelic richness (ranking)	LCA (ranking)	Climatic distance Cluster
**1**	**Ngashie-Mt Oku**	**Cameroon**	19	**1**	**1.28 (15)**	**0.14 (3)**	15	2	26.11 (15)	3.00 (10)	4
2	Lower Mann's Spring, Mt Cameroon	Cameroon	19	1	1.00 (17)	0.00 (4)	20	2	29.89 (9)	3.67 (7)	3
3	Mt Danoua	Cameroon	20	1	1.00 (17)	0.00 (4)	19	2	23.47 (19)	2.50 (12)	4
**4**	**Moka**	**Equatorial Guinea**	18	5	**1.95 (6)**	**0.14 (3)**	13	2	**27.31 (13)**	**2.83 (11)**	**3**
5	Chuka, Central province	Kenya	20	5	1.00 (17)	0.14 (3)	19	4	28.10 (11)	3.50 (8)	4
**6**	**Kinale, Central province**	**Kenya**	19	5	**2.69 (2)**	**0.43 (1)**	19	2	**30.26 (8)**	**3.33 (9)**	**1**
**7**	**Kapcherop, Cherangani Forest, Rift Valley**	**Kenya**	20	**3**	**2.46 (3)**	**0.29 (2)**	18	2	31.90 (6)	3.83 (6)	2
8	Kakamega Forest, Western Province	Kenya	20	5	1.16 (16)	0.00 (4)	17	2	35.96 (1)	4.00 (5)	4
9	Londiani, Rift Valley	Kenya	20	5	1.89 (10)	0.14 (3)	20	2	31.91 (5)	3.67 (7)	4
10	Ol Danyo Sambuk, Central province	Kenya	19	3	1.94 (7)	0.14 (3)	15	4	21.85 (23)	2.33 (13)	1
11	Taita Hills, Coast province	Kenya	20	5	1.00 (17)	0.00 (4)	18	4	20.85 (24)	2.17 (14)	1
12	Lari, Central province	Kenya	12	3	1.00 (17)	0.14 (3)	12	4	24.98 (17)	2.17 (14)	2
**13**	**Kibiri Forest, Western province**	**Kenya**	16	5	1.16 (16)	0.00 (4)	12	**2**	**35.96 (1)**	**4.50 (3)**	4
14	Marovoay	Madagascar	5	4		0.14 (3)	6	3		1.83 (16)	4
**15**	**Lakato forest**	**Madagascar**	33	**4**	**1.81 (12)**	**0.29 (2)**	25	**3**	**21.97 (22)**	**2.50 (12)**	4
16	Antsahabiraoka	Madagascar	18	4	1.00 (17)	0.14 (3)	6	3		1.67 (17)	4
17	Ngel Nyaki Forest Reserve, Nigeria	Nigeria	9	1	1.00 (17)	0.00 (4)	7	2	22.93 (20)	2.50 (12)	4
18	Mpumalanga	South Africa	19	5	1.90 (9)	0.14 (3)	18	1	14.80 (27)	3.00 (10)	2
19	KwaZulu-Natal	South Africa	17	5	1.81 (13)	0.14 (3)	12	1	17.96 (26)	1.83 (16)	1
20	Meru Catchment Reserve	Tanzania	19	3	1.97 (5)	0.14 (3)	16	4	24.66 (18)	3.00 (10)	2
**21**	**Kilimanjaro Catchment Forest Reserve**	**Tanzania**	17	**5**	**2.96 (1)**	**0.43 (1)**	11	4	27.87 (12)	2.50 (12)	2
**22**	**Kindoroko Catchment Reserve**	**Tanzania**	14	**2**	**1.00 (17)**	**0.29 (2)**	6	4		1.17 (18)	2
23	Shume Magamba Catchment Forest Reserve	Tanzania	20	5	1.77 (14)	0.14 (3)	3	4		1.00 (19)	1
24	Kidabaga	Tanzania	15	5	1.85 (11)	0.14 (3)	15	4	26.86 (14)	3.00 (10)	2
**25**	**Udzungwa**	**Tanzania**	16	5	2.05 (4)	0.00 (4)	10	**4**	**32.90 (2)**	**3.67 (7)**	2
26	Kibale Forest Natural Park	Uganda	20	1	1.00 (17)	0.00 (4)	20	2	31.69 (7)	5.17 (1)	2
27	Kalinzu Forest Reserve	Uganda	20	1	1.00 (17)	0.00 (4)	20	2	29.36 (10)	4.00 (5)	2
28	Bwindi Forest	Uganda	19	1	1.28 (15)	0.00 (4)	20	2		4.67 (2)	2
29	Mabira Forest	Uganda	20	1	1.93 (8)	0.00 (4)	20	2	32.40 (4)	4.17 (4)	2
30	Nyanga National Park	Zimbabwe	20	5	1.00 (17)	0.00 (4)	19	1	22.78 (21)	3.33 (9)	4
**31**	**Cashel Valley Chimanimani**	**Zimbabwe**	20	5	1.00 (17)	0.00 (4)	13	**1**	**25.30 (16)**	**3.00 (10)**	2
32	Chirinda Forest Reserve Chipinge	Zimbabwe	19	5	1.00 (17)	0.00 (4)	20	1	18.94 (25)	2.00 (15)	2

A subset of 32 *Prunus africana* populations, from across 9 African countries is characterized by genetic data (number of individuals, haplotype richness, allelic richness, occurrence of locally common alleles, similarity in allelic composition) and climatic data. Haplotype/allelic richness and presence of locally common alleles are ranked, with highest ranking attributed to populations with the highest value of these parameters. Priority populations for conservation are highlighted in bold.

We assembled a dataset of 1,500 georeferenced observations of *P. africana* (mostly recorded between 1965 and 2010) that cover the range of the species and enabled us to define its ecological niche and to model its potential distribution. We assembled the dataset accessing the following sources: Mpumalanga Tourism & Parks Agency Lydenburg, South Africa (botanist Mervyn Lotter), University of Bangor (John Hall), World Agroforestry Centre (ICRAF), GBIF [75] and JSTOR Plant Science [76]. For the spatial analysis, a raster size of 30 minutes (about 50 km near the equator) was used and molecular marker data were formatted in such a way to attribute coordinates to each allele/haplotype.

### Species Distribution Modeling

*P. africana*’s potential distribution was modeled using the distribution modeling program Maxent. This program uses an algorithm of maximum entropy to calculate the ecological niche of a species and to define the areas of potential natural distribution [45], [77]. The environmental values are extracted from online geo-referenced databases. A total of 21 environmental layers were included to build the potential natural distribution model under current conditions: 19 BIOCLIM variables were derived from the WorldClim database [78], [79], soil data from the World Soil Resources Coverage map [80], and environmental data from the FAO Map on Global Ecological Zones [81]. For the prediction of species distribution, the threshold “Maximum sensitivity plus specificity” with threshold = 0.180 was chosen (for details see [82]). It should be noted that weather station coverage may not be optimal and interpolated values could be particularly problematic in montane regions due to factors such as rain shadows and fine-scale varying topography. However, WorldClim constitutes the best dataset available as it includes major climate databases from many sources (e.g., Global Historical Climatology Network, FAO, WMO, CIAT, R-HYdronet, and a number of additional minor databases). The geographic observations used for modeling the potential distribution of *P. africana* were first filtered in DIVA-GIS (www.diva-gis.org) [83] to detect outliers. All occurrence records were checked for inconsistency of their coordinates with administrative area level 1 (usually states or provinces) and for extreme values in their climatic parameters (extreme values for a minimum of 3 out of 19 bioclimatic variables examined) according to the Reverse Jackknife method [84]). After the screening, three points were excluded and the final dataset included 1,500 geographic observations.

### Spatial Analysis for Selection of Priority Populations for Conservation

Two approaches to select priority populations for conservation were used. One method (S1) maximizes genetic diversity and distinctiveness of the conservation unit based on a combination of genetic and climatic criteria. A second method (S2) is based exclusively on genetic data and optimizes representativeness of the genetic diversity found throughout the species’ range. Each of the selected priority populations was further evaluated for urgency of conservation action on the basis of current protected status and the threat posed by predicted future climate conditions.

#### Method S1

We used the following steps to add populations to the list of priority sites: populations were clustered based on their genetic (chloroplast- and nuclear-based) and climatic similarity; in each genetic cluster, the population with highest ranking in allelic/haplotype richness and presence of locally common alleles was selected. The second step identified populations in different climatic clusters not represented in the selection above. In each climatic cluster, the population with the highest rank in both genetic parameters (allelic/haplotype richness and presence of locally common alleles) was added to the priorities for conservation.

1: Clustering of populations based on allelic composition: the degree of genetic similarity between populations was calculated in the R statistics environment version 2.14 [85] determining Nei’s distance [86], [87] through the R function “dist.genpop” in the package adegenet version 1.3–4 [88]. Hierarchical clustering was performed with the R function “hclust” in the package mva, version 1.0–3, using the unweighted pair-group method of arithmetic averages (UPGMA). The Kelley-Gardner-Sutcliffe penalty function for a hierarchical cluster tree was calculated using the function “kgs” in the package maptree [89] to suggest a number of clusters in the dataset. The cophenetic distance was calculated with the function “cophenetic” in the package stats. The cophenetic distance between two observations is defined as the distance (or similarity) level at which two observations become part of the same cluster [90]. With the function “cor” in the package stats, version 2.15.2, the correlation between Nei’s genetic distance and the cophenetic distance was determined to validate the clustering.

2: Identification of populations with highest allelic/haplotype richness: the dataset analyzed in this study included 147 alleles at 6 nSSR loci and 19 alleles at 7 cpSSR loci. A total of 22 multilocus haplotypes were constructed by combining single cpSSR loci. An inherent difficulty with many diversity studies arises from the well-known property that diversity of a sample increases with sample size [91], [92]. Results of analyses may thus depend on the number of samples taken within each subunit of the study area. Haplotype richness for cpSSR and allelic richness for nSSR were determined in DIVA-GIS, applying the rarefaction method to correct for sample size bias, recalculating richness measured only within subunits (30 min grid cell size) containing 7 or more trees (equivalent to 7 haplotypes or 84 nuclear allele observations) (see detailed methodology in [79]). Populations were then ranked based on their allelic/haplotype richness. The Kruskal-Wallis test [93] was carried out to test for significant differences among populations in the distribution of allelic richness.

3: Identification of populations with presence of locally common alleles: locally common alleles are those repeatedly observed but only in a small area relative to the species’ distribution; they are of high interest, especially if they are associated with adaptive traits [94], [91]. The definition considers as locally common those alleles with a frequency higher than 5% in a local population and occurring in less than 25% of all populations examined [51]. The distribution of locally common alleles was examined to further identify zones of high or unique intra-specific diversity. The average number of locally common alleles was calculated using GenAlEx 6.5 [95]. Populations were ranked based on this variable.

4: Climate clustering: *P. africana* observations were clustered based on climatic data, assuming that natural populations from different climate zones would show variable adaptive traits not captured by the analyses of SSR markers data. The 1,500 observations were clustered on the basis of 19 bioclimatic variables, extracted by point from the 2–5 minutes Wordclim dataset. The function “dist” in the R statistics environment 2.14.0 was used to calculate the Euclidian distance and hierarchical clustering was carried out using the UPGMA method. As for the clustering above, based on genetic similarity, we used the Kelley-Gardner-Sutcliffe penalty function, in order to derive an optimal number of clusters, and the cophenetic correlation coefficient to validate the clustering.

#### Method S2

The approach S2 was based on the identification of a minimum number of populations needed to include all the genetic diversity based on both chloroplast and nuclear markers. The procedure adopted in DIVA-GIS is called ‘r*eserve selection’.* It generates a selection of grid cells (30 minute cell size) that are complementary to each other in terms of diversity included in each cell, and that captures the maximum amount of diversity in the smallest number of cells possible (see [96]). The algorithm also identifies priorities, indicating a ranking for the geographic units of interest. The first population chosen has the highest allelic richness; each successive population selected best complements the intra-specific diversity already represented within the previously selected priority populations. The ‘*reserve selection*’ algorithm, developed by Rebelo and Sigfried [96], was applied to a combined dataset, including chloroplast and nuclear molecular markers from across the 32 sample populations studied. This procedure enabled a selection of cells/populations not only based on their diversity, but also on differences/complementarity in allelic composition.

### Conservation and Threats

The modeled potential distribution was combined with data on the location of protected areas. The portion of *P. africana*’s potential distribution found within protected areas was determined in order to derive an indicator for the *in situ* conservation status of the species. The World Database of Protected Areas (WDPA) [97] was used to calculate the proportion of *P. africana*’s potential distribution that falls within the boundaries of protected areas of different types. WDPA includes detailed information on flora, fauna, and a wide range of climatic, environmental and socioeconomic data for 741 protected areas across 50 countries. More detailed results were presented for three countries (Kenya, Uganda and Tanzania) that host high genetic diversity for *P. africana*, in order to examine more closely specific threats, such as climate change and land conversion to croplands.

Loss of forest cover in Africa has been substantial over the past 20 years, with an average area of ca. 3.7 million ha/year converted to other land uses in the period 1990–2010 [98]. The Global Land Cover Map 2000 [99] was used to identify those areas with only natural vegetation, excluding “croplands” (regions with over 50% crop fields or pasture, equivalent to intensive cultivation and/or sown pasture) and other land uses (e.g., urban settlements).

The potential threats from changes in climatic conditions at the regional scale were also assessed, by comparing the potential distribution of *P. africana* under the current climate, based on the species’ current distribution, with the potential distribution under future climatic conditions. Future climate projections were developed for 2050 under the A2 emission scenario (with constantly increasing emission rate) from the average of three different Global Circulation Models (GCMs) downloaded from the GCM Data Portal [100]: CCCMA-CGCM3.1-T63, HCCPR HADCM3 and CSIRO-MK3.0. Finally, to determine the potential distribution of *P. africana* during the peak of the last glacial period (between 26,500 and 19,000–20,000 years ago) the GCM “CCSM: Last glacial maximum (LGM; ∼21,000 years BP)” downloaded from WORLDCLIM (http://www.worldclim.org/past) was used [Source: Paleoclimate Modeling Intercomparison Project Phase II (PMIP2)].

## Results

### Distribution Range of *Prunus africana* and Spatial Analysis of Genetic Diversity

**Figure 1:**
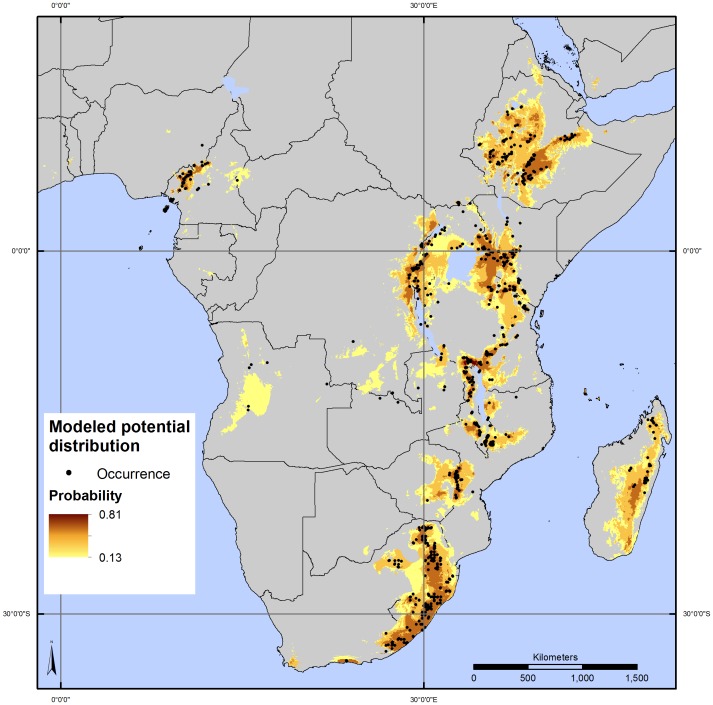
*Prunus africana* observations and modeled potential distribution. Probability of occurrence of *P. africana* is determined on the basis of climatic/environmental parameters and indicated by different colors, from dark brown (high probability) to yellow (low probability).

Based on our dataset of 1,500 georeferenced observations, the presence of *P. africana* has been directly recorded in 22 African countries, while the modeled distribution extends to 34 countries (Fig. 1).

**Figure 2:**
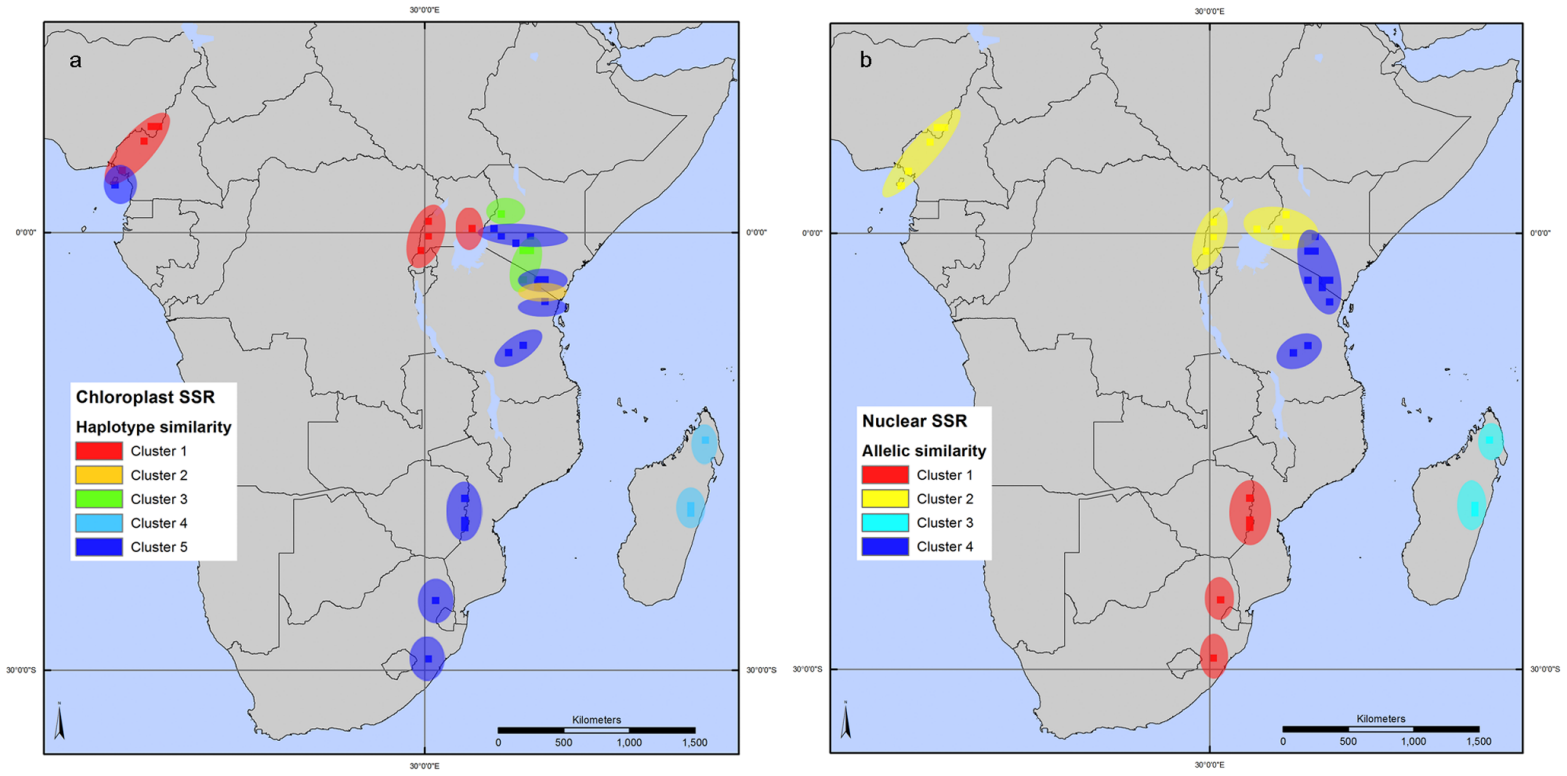
Clustering of *Prunus africana* populations based on molecular marker data. The 32 populations, represented by 30 minute grid cells, are grouped by Nei’s distance, based on similarity of haplotypes (cpSSR) (2a) and similarity of nuclear microsatellite (nSSRs) allelic composition (2b).

For the 32 populations sampled for genetic analyses, the clustering by similarity of allelic profile revealed 5 and 4 groups, respectively for chloroplast-based (Fig. 2a) and nuclear-based (Fig. 2b) SSRs. The cophenetic correlation coefficients for cpSSRs and nSSRs were 0.79 and 0.73, indicating a good clustering structure in each case. The clustering results converged, showing that Madagascan populations are distinctive, and highlighting a clear separation between East and West African populations. Based on cpSSRs, these western populations showed a similar genetic profile to those found in Uganda (Fig. 2a). Populations from Zimbabwe and South Africa grouped with populations from Kenya and Tanzania; Kenya included populations from 2 clusters, while Tanzania included populations from 3 clusters (Fig. 2a).

Based on nSSRs, the grouping produced slightly different results: the western populations showed a similar genetic profile to those found in Uganda and western Kenya (Fig. 2b). Populations from Zimbabwe and South Africa clustered together and formed a separate group from populations in Kenya and Tanzania (Fig. 2b). Kenya included populations from 2 clusters, while Tanzania included populations from 1 cluster.

**Figure 3:**
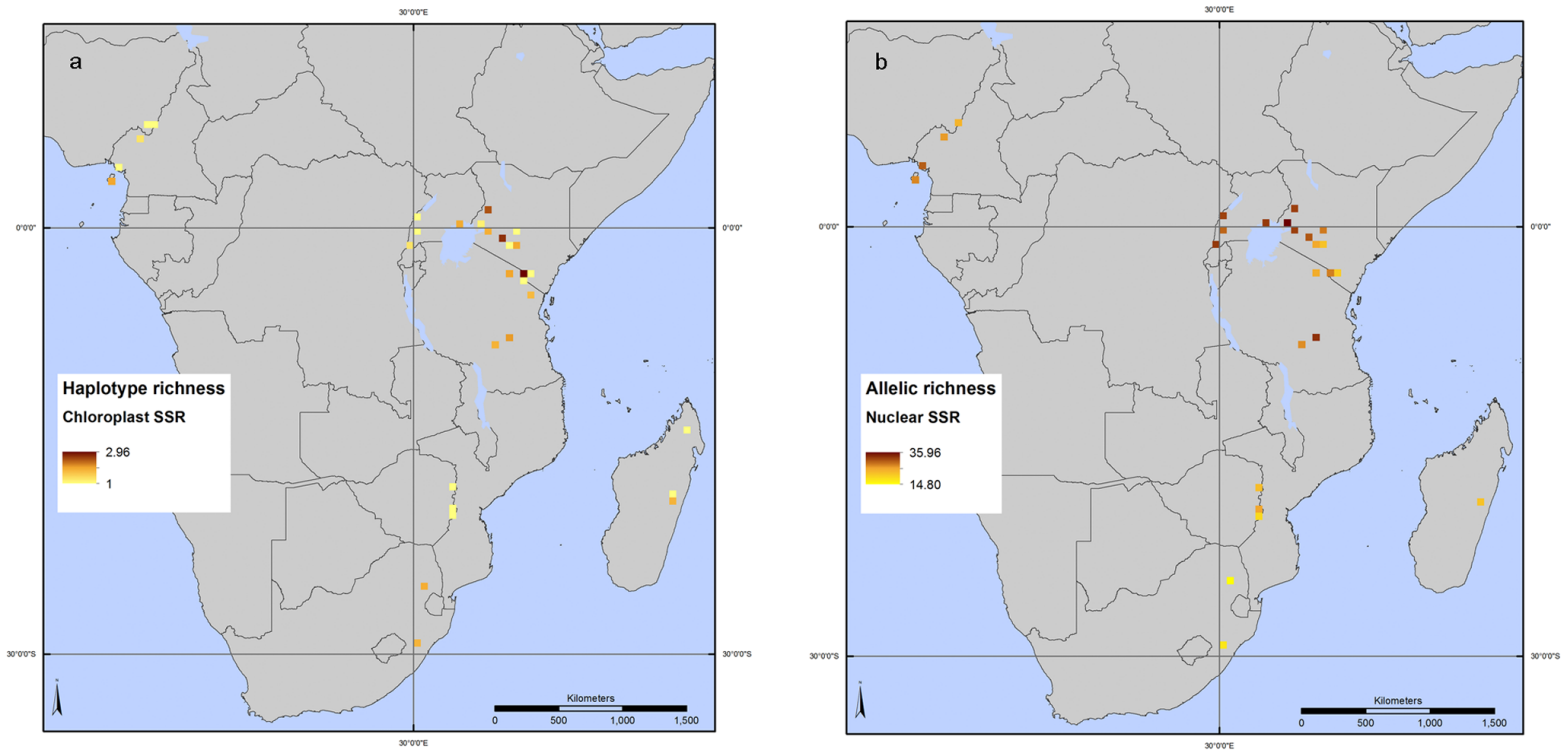
*Prunus africana* haplotype richness and allelic richness. Haplotype (cpSSR) (3a) and allelic (nSSR) (3b) richness are determined for 32 populations, after rarefaction, using a 30 minute grid cell size.

The spatial distributions of haplotype (chloroplast SSRs) richness and allelic (nuclear SSRs) richness were determined after rarefaction (Figure 3 a,b). Both types of markers point to populations in East Africa as the ones with the highest allelic richness. The Kruskal-Wallis test (p-value < 0.0005) showed that the 32 populations had significantly different distributions of allelic richness.

The following populations had highest haplotype richness, in descending order (Table 1): pop. No. 21 (Kilimanjaro catchment, Tanzania), 6 (Kinale, Kenya), and 7 (Cherangani Forest, Kenya). Highest ranking populations for nuclear allelic richness do not overlap with those having highest haplotype richness. They are pop. No. 8 (Kakamega Forest, Kenya), 13 (Kibiri Forest, Kenya), and 25 (Udzungwa, Tanzania). Populations with locally common alleles were located primarily in Kenya and Uganda, but also in Tanzania and Madagascar (Table 1).

The set of priority populations that combined highest value of haplotype richness and highest presence of locally common alleles, in each of the 5 clusters identified, based on chloroplast markers, was the following in ascending cluster number (Table 1): pop. No. 1 (Ngashie - Mt Oku, Cameroon), 22 (Kindororo Catchment reserve, Tanzania), 7 (Cherangani Forest, Kenya), 15 (Lakato Forest, Madagascar), 21 (Kilimanjaro catchment, Tanzania). The set of priority populations that combined highest value of allelic richness and highest presence of locally common alleles, in each of the 4 clusters identified based on nuclear markers, was the following: pop. No. 31 (Cashel Valley Chiamanimani, Zimbabwe), 13 (Kibiri Forest, Kenya), 15 (Lakato Forest, Madagascar), 25 (Udzungwa, Tanzania). One of the priority populations above overlapped: 15 (Lakato Forest, Madagascar).

After the selection above, additional populations were included in the priority list based on the analysis of climatic variables across the sites where the species is found. Those populations occurring in areas having unique climatic conditions, not selected based on the previous criteria, were added among the priorities for conservation. The rationale for this is that in the absence of quantitative genetic data, distinctive environmental conditions can be a proxy for useful adaptive variation.

**Figure 4:**
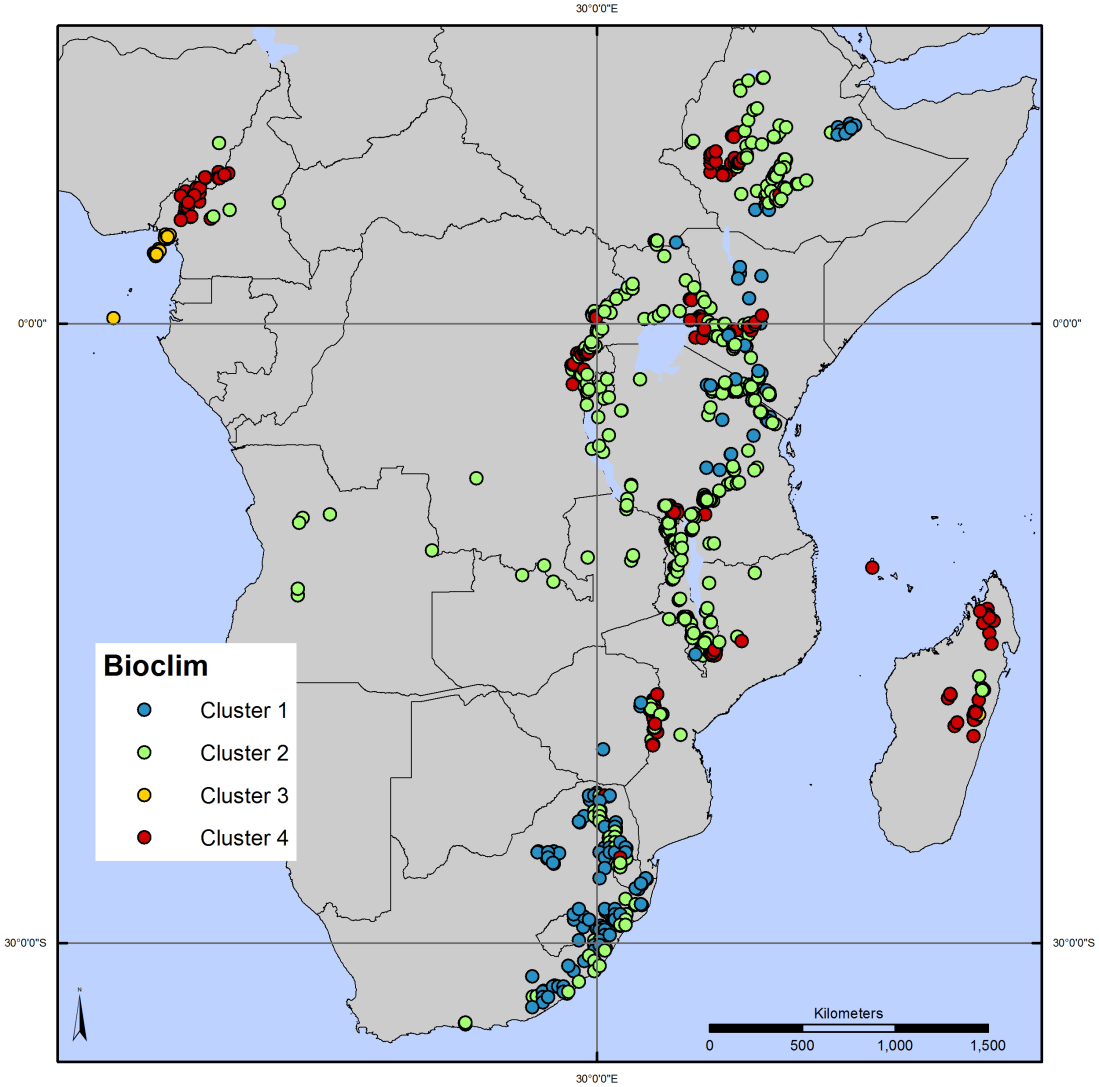
Clustering of 1,500 *Prunus africana* observations based on level of similarity of bioclimatic variables. Bioclimatic values for 19 variables were associated with all *P. africana* records. Bioclimatic values were extracted from 2.5 minute rasters obtained from the Worldclim website. The observation points are grouped (each cluster is highlighted with a different colour) by Euclidean distance.

A total of 4 distinct climate clusters were identified using all *P. africana* occurrence observations available (Fig. 4). The cophenetic correlation coefficient obtained was 0.81, confirming the validity of the method adopted. While clusters 1, 2 and 4 correspond to climatic conditions with a broad distribution across the species range, and include a large number of the individual observations, cluster 3 characterizes a limited area, with very distinct climatic features (low seasonality in temperature and high annual precipitation, between 2,400 and 3,000 mm) (marked in yellow in Fig. 4). Of the 32 populations for which genetic data were available, populations in climate clusters 1 and 3 were not represented in the selection based on genetic parameters. Thus, in each of these two clusters, the population with highest haplotype/allelic richness and presence of locally common alleles was added to the selection of priority populations for *P. africana* conservation (Table 1). The populations added were the following: No. 6 (Kinale, Kenya), and No. 4 (Moka, Equatorial Guinea).

The adoption of approach S1 to select priority areas generated a list of 10 priority populations (highlighted in bold on the left hand side of Table 1), that would maximize inclusion of the genetic and climatic diversity measured across the 32 populations sampled. Six of the 10 priority populations are located in Kenya (3) and Tanzania (3). The others are in Madagascar (1), Cameroon (1), Equatorial Guinea (1) and Zimbabwe (1).

The S2 approach generated a list of 19 priority populations presented in Table 2. Two of the original 19 priority populations, in Kakamega and Kibiri forests, fall within the same grid cell due to their closeness, and are treated as one population; the final number of priority populations is 18. Seven of the 18 priority populations are located in Kenya, followed by Madagascar (3), with 2 each in Tanzania (2), Zimbabwe (2) and Uganda (2), and with one each in Equatorial Guinea (1) and Cameroon (1). Being focused on representativeness of the genetic diversity found throughout the species’ range, approach S2 selects a larger number of sites among the priorities, and includes also one of the two populations characterized by peculiar climatic conditions (pop. No. 4 in Equatorial Guinea).

**Table 2:** *Prunus africana* populations of conservation priority based on the second selection method proposed (S2).

			Both SSR
Code	Name of population	Country	Reserve selection (priority)
8	Kakamega Forest, Western Province	Kenya	1
13	Kibiri forest, Western province	Kenya	1
6	Kinale, Central province	Kenya	2
31	Cashel Valley Chimanimani	Zimbabwe	3
15	Lakato Forest	Madagascar	4
28	Bwindi Forest	Uganda	5
5	Chuka, Central province	Kenya	6
20	Meru Catchment Reserve	Tanzania	7
22	Kindoroko Catchment Reserve	Tanzania	8
4	Moka	Equatorial Guinea	9
29	Mabira Forest	Uganda	10
12	Lari, Central province	Kenya	11
10	Ol Danyo Sambuk, Central province	Kenya	12
16	Antsahabiraoka	Madagascar	13
30	Nyanga National Park	Zimbabwe	14
3	Mt Danoua	Cameroon	15
9	Londiani, Rift Valley	Kenya	16
11	Taita Hills, Coast province	Kenya	17
14	Marovoay	Madagascar	18

Priorities are identified within 32 *Prunus africana* populations for which genetic data are available. The method is based on the ‘*reserve selection*’ analysis carried out in DIVA-GIS. The method is aimed at enhancing complementary of the genetic diversity represented within the populations selected for conservation priority. The 18 populations selected for conservation priority are listed (2 of the original populations fall within the same grid cell due to their closeness, therefore are treated as one population and have the same ranking).

A combination of the two approaches allows a further selection of 6 priority populations, which were identified as priorities using both methods. These populations constitute a core set of proposed conservation areas: 2 populations in Kenya (pop. No. 6, 13), and one population each in Equatorial Guinea (pop. No. 4), Madagascar (pop. No. 15), Tanzania (pop. No. 22), and Zimbabwe (pop. No. 31). Of the 32 populations sampled for genetic analyses, 21 are included within official conservation areas, and 4 others are in sites proposed for special protection (Table 3). An assessment at the pan-regional level, indicates that protected areas cover 39% of the observed occurrences of *P. africana* and 16.7% of its potential current distribution. Among the 6 populations that are selected as priorities by both approaches, 4 are within protected areas.

**Table 3:** Current conservation status and expected modeled future climate suitability (2050) for 32 *Prunus africana* populations, across 9 African countries.

Coder	Name of population	Country	WDPA protected areas	IUCN Category	Modeled Climate 2050
1	Ngashie-Mt Oku	Cameroon	Proposed		suitable
2	Lower Mann's Spring, Mt Cameroon	Cameroon	Proposed		suitable
3	Mt Danoua	Cameroon	Proposed		suitable
**4**	**Moka**	**Equatorial Guinea**	Designated	Ib	suitable
5	Chuka, Central province	Kenya	Designated	II	suitable
**6**	**Kinale, Central province**	**Kenya**	NOT PROTECTED		suitable
7	Kapcherop, Cherangani Forest, Rift Valley	Kenya	Designated		suitable
8	Kakamega Forest, Western Province	Kenya	Designated		MARGINAL
9	Londiani, Rift Valley	Kenya	NOT PROTECTED		suitable
10	Ol Danyo Sambuk, Central province	Kenya	Designated	II	suitable
11	Taita Hills, Coast province	Kenya	Designated		NOT SUITABLE
12	Lari, Central province	Kenya	NOT PROTECTED		suitable
**13**	**Kibiri forest, Western province**	**Kenya**	Designated		MARGINAL
14	Marovoay	Madagascar	NOT PROTECTED		suitable
**15**	**Lakato forest**	**Madagascar**	NOT PROTECTED		suitable
16	Antsahabiraoka	Madagascar	NOT PROTECTED		suitable
17	Ngel Nyaki Forest Reserve, Nigeria	Nigeria	Designated		MARGINAL
18	Mpumalanga	South Africa	Designated		suitable
19	KwaZulu-Natal	South Africa	Designated	IV	suitable
20	Meru Catchment Reserve	Tanzania	Designated	II	suitable
21	Kilimanjaro Catchment Forest Reserve	Tanzania	Designated	II	suitable
**22**	**Kindoroko Catchment Reserve**	**Tanzania**	Designated		MARGINAL
23	Shume Magamba Catchment Forest Reserve	Tanzania	Proposed	IV	suitable
24	Kidabaga	Tanzania	Designated	IV	suitable
25	Udzungwa	Tanzania	Designated	II	MARGINAL
26	Kibale Forest Natural Park	Uganda	Designated	II	suitable
27	Kalinzu Forest Reserve	Uganda	NOT PROTECTED		suitable
28	Bwindi Forest	Uganda	Designated	II	suitable
29	Mabira Forest	Uganda	NOT PROTECTED		NOT SUITABLE
30	Nyanga National Park	Zimbabwe	Designated	II	suitable
**31**	**Cashel Valley Chimanimani**	**Zimbabwe**	Designated		suitable
32	Chirinda Forest Reserve Chipinge	Zimbabwe	Designated		suitable

Populations highlighted in bold are those selected for conservation priority based on both selection approaches (S1 and S2) presented in this study. Sites that are not officially protected or are expected to present future climate conditions unsuitable for *P.africana* are highlighted in capital letters, together with areas falling at the margin of the modeled distribution under future climate scenario in 2050. For some protected areas the IUCN category is not available.

**Figure 5:**
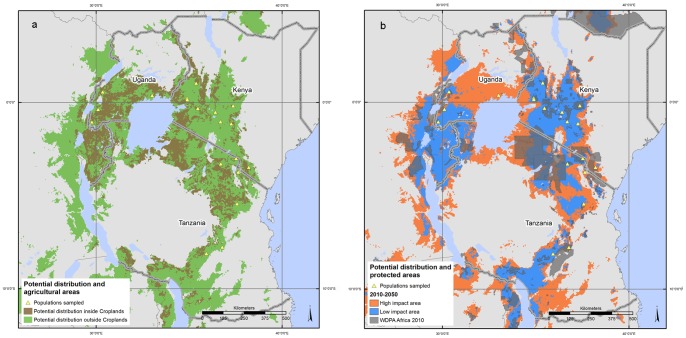
*Prunus africana* modeled potential distribution in Kenya, Tanzania and Uganda with respect to croplands and protected areas. *P. africana* modeled potential distribution is shown with respect to areas occupied by >50% croplands (5a), and to the location of protected areas (5b). Areas with expected high and low impact of climate change in 2050 are also highlighted (5b). In low impact areas (blue), no changes in species distribution are expected, while in areas of high impact (red), climatic conditions are expected to become unsuitable for *P. Africana.* The location of 19 populations, for which genetic data are available, is also shown.

However, a closer look at 3 countries (Kenya, Uganda and Tanzania) where the sampled *P. africana* populations present the highest haplotype and allelic richness, revealed that a considerable portion of the area with suitable climate for *P. africana* is not covered by natural vegetation; large parts of it have been converted to cropland (Fig. 5a). Natural vegetation areas correspond to just 21.5% of the species’ potential distribution; of this fraction, the portion covered by protected areas corresponds to 20.5% (Fig. 5b). This means that only ca. 4% of the potential distribution of the species in Kenya, Uganda and Tanzania is inside protected areas.

**Figure 6:**
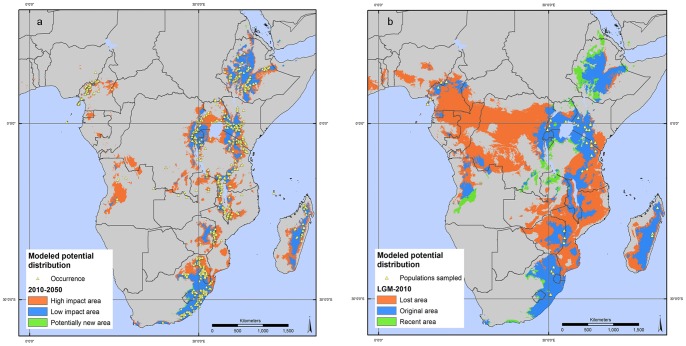
*Prunus africana* modeled potential distribution under past, current and future conditions in 2050. (6a) The spatial distribution of all *P. africana* observation points is shown. Areas in red are expected to be highly affected by future climate change; in low impact areas (blue) no changes in species distribution are expected; areas in green are expected to become suitable for *P. africana*. (6b) The past scenario refers to the last glacial maximum (LGM), about 21,000 years before present. Blue indicates areas with continued habitat suitability since LGM until present (original areas). Green indicates areas most likely unsuitable for *P. africana* at the LGM, but suitable at present (recent areas of expansion). Red represents areas suitable during LGM but no longer suitable at present (lost areas). The spatial distribution of the 32 sampled populations, for which genetic data are available, is indicated by yellow triangles.

The predicted suitable habitat for the species, according to climate scenarios based on average values of three GCMs, is presented in Fig. 6a. This analysis indicated that a considerable portion (53%) of the current range is expected to become unsuitable for *P. africana* by year 2050 (red areas), as a result of changing climate, with large portions of modeled distribution disappearing from the map (e.g., potential range in Angola) and a very modest expansion of the species to new suitable habitats (1% of area expected to be occupied by the species in 2050), while the blue areas indicate continued suitability for the next 40 years.

The modeled distribution of *P. africana* in 2050 in Kenya, Uganda and Tanzania is predicted to be impacted by climate change (Fig. 5b), and the range of suitable habitats is expected to decrease by 54% from 2010 to 2050; the part of the range included in current protected areas is expected to shrink by 46% by 2050.

The current modeled potential distribution of *P. africana* was compared with the modeled species distribution during the last glacial maximum, ca. 21,000 years ago (Fig. 6b). The range is estimated to have shrunk by approximately 45%; on this basis, likelihood of future losses can be estimated.

The modeled climate for 2050 in each sampled population is reported in the last column of Table 3.

For two populations, No. 11 in Kenya and No. 29 in Uganda, the future climatic conditions are predicted to become unsuitable for *P. africana*. Another five populations (pop. No. 8 and 13 in Kenya, No. 22 and 25 in Tanzania, No. 17 in Nigeria) will be located at the margin of the modeled distribution in 2050. Of the 6 priority populations, 2 will be located at the margin of the modeled distribution: No. 13 in Kenya, and No. 22 in Tanzania.

## Discussion

Both approaches presented (S1 and S2) to select priority populations indicate a high conservation priority for populations in the eastern part of the distribution of *P. africana*, particularly in Kenya and Tanzania, which harbor a large portion of the genetic diversity found across the species’ range. At a country level, Kenya has unique opportunities to contribute to the conservation of the species, as discussed also in Muchugi et al. [101].

The patterns of genetic variation found in *P. africana* [40], [42], [101], [102] are associated with the Afromontane habitats occupied by the species, which play the role of islands of genetic diversity [58]. The slight differences in clustering of populations based on the two types of markers used (nuclear or chloroplast SSRs) may be explained by the fact that cpDNA markers tend to reflect gene flow patterns that are more historically remote than the nuclear markers [103]. The Rift Valley disjunction - Albertine or eastern branch depending on the type of marker used - appears to have caused a major barrier between eastern and western populations [40] and explains the relatedness of populations from Cameroon, Uganda and Western Kenya, which are quite different from those found in central Kenya. In addition, it is clear from genetic analyses that populations from Madagascar are distinct and highly diverse [40], [42], [101]. Populations in Cameroon and Equatorial Guinea, although not quite as diverse as those mentioned above, are also important as their environmental conditions diverge sufficiently to almost certainly have given rise to variation in genes controlling adaptive traits.

Obtaining genetic information specific to valuable traits requires considerable time and cost but, as genomic tools develop, their potential for describing useful variation of expressed genes will likely be a breakthrough for conservation genetics [104]. The approach for selecting priority populations for conservation presented here is based on neutral molecular markers which are extremely useful for discerning gene flow and evidence of historic events such as genetic bottlenecks [105] and are a useful basis to define conservation priorities. Populations with high diversity in neutral markers can be considered suitable candidates for high adaptive variation as well. In addition, disjunctions in the distribution range (like in the case of *P. africana*) are indicative of isolation and we might expect to find local adaptive variation on this basis.

The approach proposed enables a reduction of the number of priorities to a minimum set of core sites optimally distributed across the range of *P. africana*. In addition, the combination of the two methods described (S1 and S2) allows inclusion within priorities of those populations with highest genetic diversity across genetically separate clusters, but also of populations with lower diversity belonging to distinct climate clusters, potentially harbouring important adaptive properties. The clustering and ranking were obtained through a user friendly sequence of steps, with the support of freely available software, enabling conditions for a wide uptake.

The results reveal that although the species is not in danger of extinction, some important populations, with distinct characteristics, are threatened and their loss would reduce the livelihood potential for local people. Populations in Cameroon and Madagascar have been exposed to sustained high rates of exploitation [61], [63]. Bark extraction has been also high, but less intensive, in Kenya and on the island of Bioko (Equatorial Guinea) [61], [106]. Debarking of *P. africana* often occurred within Afromontane forest habitats of global conservation significance including in protected areas [107], [108] and unpublished reports indicate that harvest still occurs in such areas (pers. comm. with stakeholders). In addition, poor natural regeneration has been observed in some locations due to unsuitable conditions for establishment, attributable to insufficient light reaching the forest floor and the accumulation of thick litter of competing exotic species [109].

Only 4 *P. africana* populations of priority conservation value based on both approaches (S1 and S2) are found within the boundaries of protected areas. These populations constitute a starting point for a conservation strategy for *P. africana* at the continental scale, because conservation actions would be facilitated by the existing conservation measures in place. However, information on the respective IUCN conservation status is not available for all the identified priority sites for *P. africana* conservation; therefore it is not clear what level of protection is applied, and there are concerns about the effectiveness of protection despite the legal status.

Illegal logging and other types of encroachment into protected areas and fire frequently pose threats to the species. The situation might be worsened by a combination of different pressures on the forest cover. For example in Zimbabwe, Jimu [110] has reported that the introduction of plantations of exotic *Pinus, Eucalyptus* and *Acacia* spp. has changed the landscape of the Afromontane region considerably, and increased fire susceptibility in the forest cover. These commercial species also encroach on protected areas and compete strongly with other tree species, including *P. africana*. Clearance for agriculture is a major threat affecting forested areas where *P. africana* occurs, as illustrated for the eastern part of the range of the species. It is not clear how climate change and further expansion of agriculture will interact with threats in different parts of the Afromontane regions, where *P. africana* would be pushed to higher elevations.

Potential threats from climate change in 2050 are envisaged for some highly diverse populations in Kenya, Tanzania and Uganda. The projections presented in this paper do not account for phenotypic plasticity, which affects the way a species responds to a change in environmental conditions. However, they are congruent with those generated by similar studies limited to East African countries [111], [112], although these were based on different approaches (e.g., maps of vegetation types used as a proxy for the distribution of specific woody species, different time scale and future climate scenarios chosen). All predictions indicate a future contraction of the area suitable for *P. africana,* with the degree of reduction highly variable depending on the climate scenarios and approaches adopted.

It is not clear how populations in Cameroon, Equatorial Guinea, Nigeria, Angola and South Africa will respond to environmental challenges. They may be less well equipped as they have reduced levels of genetic diversity, probably attributable to their greater distance from the centre of origin of the species. On the other hand, trends are unclear; more peripheral lowland populations may contain more useful traits for climate change adaptation than other populations occurring at higher elevations. As an example, recommendations for *Pinus oocarpa* populations in Mexico suggest transferring seed at higher altitudes upwards, following a progressive change in climatic conditions [113].

A broad corridor was revealed by modeling the potential range of the species in the past; it connected Uganda with the Democratic Republic of the Congo and further with West African countries, like Cameroon and Nigeria. This may explain the current occurrence of the species and the possible migration route, considering the similarities found in the genetic profile of West African populations and those located on the western side of the Rift valley in eastern Africa [40], [101]. It would be useful to carry out new genetic studies in the areas of recent expansion of the species, to understand how genetic diversity in the species relates to past refugia [114]. In addition, important collection gaps exist in Ethiopia and Angola and future studies should include these countries.

## Final Considerations

The priority areas for conservation of *P. africana* identified in the present study include genetically unique and highly diverse *P. africana* populations. The areas selected are also representative of the main climatic conditions found across the species range and constitute a network of priority sites for conservation of *P. africana* across its range. Gap analyses at a finer scale should be carried out to identify areas with particular environmental conditions and elements of intraspecific diversity in *P. africana* not adequately represented inside the network of protected areas [52].

Encroachment and conversion of forest land to other uses threatens viability of isolated populations. Thus it is crucial to maintain a minimum population size in community forests or in a series of patches linked by pollinators in farmland. Preliminary results on gene flow in *P. africana* populations are available [115]–[117] and there is increasing evidence of how diversity and population sizes of threatened tree species, important for the livelihood of rural communities, could be maintained through the incorporation of these species in agricultural landscapes [118], [119].

The present study highlights priority populations to be considered for inclusion in a core set of *in situ* conservation units for *P. africana*, spread across the range and representing the variety of conditions in which the species grows. Particular attention should be paid to those priority sites within protected areas but expected to be in marginal conditions by 2050, due to predicted climatic changes (No. 13 in Kenya, No. 22 in Tanzania). *In situ* priority populations should be inventoried in terms of area and number of individuals, assessment of regeneration success and effective population sizes, phenological observations, biotic and abiotic factors that affect regeneration, and potential factors that could threaten individual populations.

Planting *P. africana* has been widely adopted by small-holder farmers in some countries such as Cameroon, where the intensive exploitation of the species started earlier, in the 1970s, and where only scattered remnants of natural populations can be found [120]. Land security is the major factor that enables planting to take place, and the main incentive to planting *P. africana* has been income, while the most important constraints are lack of good planting materials, destruction by animals and fire, low sale prices and lack of fertilizers as indicated in a recent survey on farmers’ decisions [120]. The main driver for the recent overexploitation of *P. africana* has been the international trade of its bark extract, increasingly monitored and subjected to regulation through application of quotas to quantities of bark extract exported from African countries. However, a growing interest for ethnoherbal remedies and a search for novel products from traditional medicinal plants have been documented in local markets; in Kenya, *P. africana* is among the most popular species traded in the local herbal industry [121]. This indicates that the exploitation pressure on the species is likely to persist, beyond the trends in the international market, therefore conservation interventions and sustainable exploitation measures need to be applied.

Attempts have been made to identify superior populations with regard to the chemical composition of the bark [122]. A molecular phylogeographic pattern was reflected in the spatial variation of certain bark constituents, such as ursolic acid. In addition, a very high concentration of the studied constituents was found in Madagascan populations, genetically distinct from the African mainland. Despite the pronounced variation in the concentration of selected bark constituents among populations, the findings did not reveal a very distinct geographical pattern in the concentration of bark constituents, therefore further investigations would be needed to cast light on these aspects. In addition, a closer dialogue with pharmaceutical companies is needed to understand better what compounds determine the medicinal properties of bark extracts.

*Ex situ* conservation efforts should be coupled with *in situ* conservation, giving priority to threatened populations with known highest genetic diversity (e.g., Kenya and Tanzania), or to the most isolated populations that consist of more than 500 mature individuals. Tests on seeds of *P. africana* have shown that seeds can tolerate desiccation under appropriate storage conditions [123]. The selection of seed sources should take into account the challenges posed by climate change [124] and common garden experiments should be established to examine the variation in adaptive traits and phenotypic plasticity. The current range of climatic conditions in which the species grows provides additional information on potential adaptation to future conditions. In addition, genomic markers may soon be available, if genes can be linked to adaptive responses. Results would be particularly important for defining planting zones in changing climates, though constraints in implementing these actions would come from the long-term financial implications and land tenure issues.
